# Structural basis for the second step of group II intron splicing

**DOI:** 10.1038/s41467-018-06678-0

**Published:** 2018-11-08

**Authors:** Russell T. Chan, Jessica K. Peters, Aaron R. Robart, Timothy Wiryaman, Kanagalaghatta R. Rajashankar, Navtej Toor

**Affiliations:** 1 Department of Chemistry and Biochemistry, University of California, San Diego, La Jolla, CA 92093 USA; 2 NE-CAT and Department of Chemistry and Chemical Biology, Cornell University, Argonne National Laboratory, Argonne, IL 60439 USA; 3 Present Address: Department of Biochemistry, West Virginia University, Morgantown, WV 26506 USA

## Abstract

The group II intron and the spliceosome share a common active site architecture and are thought to be evolutionarily related. Here we report the 3.7 Å crystal structure of a eukaryotic group II intron in the lariat-3′ exon form, immediately preceding the second step of splicing, analogous to the spliceosomal P complex. This structure reveals the location of the intact 3′ splice site within the catalytic core of the group II intron. The 3′-OH of the 5′ exon is positioned in close proximity to the 3′ splice site for nucleophilic attack and exon ligation. The active site undergoes conformational rearrangements with the catalytic triplex having different configurations before and after the second step of splicing. We describe a complete model for the second step of group II intron splicing that incorporates a dynamic catalytic triplex being responsible for creating the binding pocket for 3′ splice site capture.

## Abstract (web summary)

Group II introns are large, self-splicing RNAs that catalyze their own excision from pre-mRNA molecules. Here the authors determine the 3.7 Å crystal structure of the group II intron in the stage immediately before the second step of splicing and present a complete model for the second step of group II intron splicing.

## Introduction

**Fig. 1:**
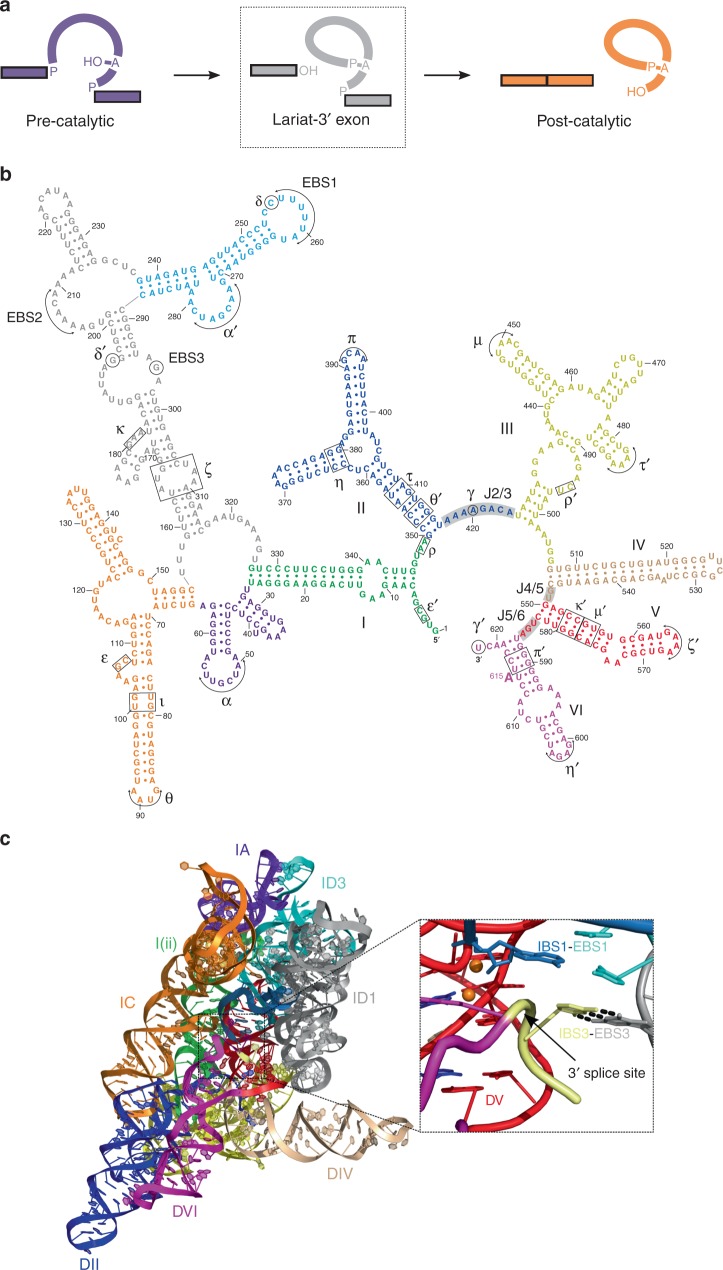
Structure of the lariat-3′ exon (pre-2s) intermediate. **a** Pathway for group II intron splicing. The bulged adenosine residue attacks the 5′ splice site in the first step of splicing to form the lariat-3′ exon intermediate. The 3′-OH of the 5′ exon then attacks the 3′ splice site in the second step that results in exon ligation. The dashed rectangle indicates the state which is captured in the pre-2s structure discussed in the present study. **b** Secondary structure of the *P.li.LSUI2* group II intron. Six domains emanate from a central wheel and are numbered I through VI. Different colors correspond to each of the domains. **c** The overall structure of the lariat-3′ exon intermediate. Domains are colored and numbered as shown in the secondary structure in Fig. 1b. Inset image shows the intact 3′ splice site positioned in close proximity to the catalytic two-metal-ion center (orange spheres) and the 3′-OH nucleophile of 5′ exon. This state directly precedes the second step of splicing with the 3′-OH poised to engage in nucleophilic attack upon the 3′ splice site. Domain VI (purple) and the IBS3-EBS3 interaction serve to properly position the 3′ splice site in the active site. These interactions result in the 3′ splice site backbone being distorted to present the scissile phosphate to the nucleophile in the second step of splicing

Group II introns are self-splicing ribozymes that catalyze two transesterification reactions to excise themselves from pre-messenger RNA. In the first step of splicing, the 2′-OH of a bulged adenosine residue is used as the nucleophile to attack the 5′ splice site (Fig. 1a). This is followed by a second step in which the free 3′-OH of the 5′ exon attacks the 3′ splice site to form ligated exons. Recently, structures of the spliceosome were determined at high-resolution using cryo-electron microscopy (cryo-EM) and revealed that its active site architecture is highly conserved with that of group II introns, with both utilizing a two-metal-ion mechanism to catalyze RNA splicing^1–4^. The spliceosomal U2-U6 snRNA pairing is analogous to domain V in group II introns with these regions forming the active site that binds the catalytic metal ions. Specifically, both U2-U6 and domain V contain a highly conserved AGC catalytic triad, which forms base triples with other nucleotides to form a triple helix known as the “catalytic triplex”. This catalytic triplex forms the binding pocket for the two active site magnesium ions. Furthermore, the spliceosomal branch-site helix contains the bulged adenosine nucleophile that is also found in domain VI of group II introns. The phylogenetic distribution, mechanistic similarities, and structural homology between the two systems suggests that group II introns are ancestral to the eukaryotic spliceosome.

Structures are currently available of the group II intron and the spliceosome at different stages of catalysis; however, the intact 3′ splice site has not yet been visualized in either system. The precise location of the 3′ splice site would provide insight into the mechanism of the second step of splicing. In group II introns, the 3′ splice site is positioned in the core via domain VI and the IBS3-EBS3 interaction^5^. Domain VI (magenta color in Fig. 1b) is covalently connected to the 3′ splice site as this is the last domain of the intron before the start of the 3′ exon. IBS3-EBS3 consists of a single Watson-Crick pair between the first nucleotide of the 3′ exon (Intron Binding Site 3, IBS3) and an internal intron base (Exon Binding Site 3, EBS3), while domain VI is covalently attached immediately upstream of the 3′ splice site. The 3′ splice site is also known to be positioned by a single Watson-Crick pair between the 3′ terminal nucleotide of the intron and a residue from the junction between domains II and III (J2/3), which is designated as the γ-γ′ interaction^6^. This interaction is critical for placing the scissile phosphate of the 3′ splice site near the catalytic metal ions.

Here we report the 3.7 Å crystal structure of a eukaryotic group IIB intron from the brown algae *Pylaiella littoralis* (*P.li.LSUI2*) in the lariat-3′ exon form with an intact 3′ splice site docked into the catalytic core. Combined with biochemical data, we compare the structure of the lariat-3′ exon with other group II intron structures to develop a model for the second step of RNA splicing.

## Results and Discussion

### Intact 3′ splice site docked into catalytic core

This crystal structure (*R*_free_ = 25.3%; *R*_work_ = 21.7%) represents the catalytic state of the group II intron immediately preceding the second step of splicing (pre-2s) (Figure 1c and Table 1). The pre-2s state is most similar to the spliceosomal P complex^7–9^, however currently there is no structure of the spliceosome at this stage of catalysis with an intact 3′ splice site. The intact 3′ splice site is aligned in the active site adjacent to the two catalytic Mg^2+^ ions and the 3′-OH nucleophile of the 5′ exon. The intact 3′ splice site was biochemically confirmed with a sequence analysis of the crystallized RNA (Supplementary Figure 1). The presence of iridium hexammine promotes exon ligation (Supplementary Figure 2), whereas its absence stalls splicing in crystallo prior to the second step (Supplementary Note 1). In addition, the post-catalytic structure^10^ (iridium hexammine derivative) was further refined (*R*_free_ = 25.5%; *R*_work_ = 20.8%) (Table 1).

**Table 1:** Data collection and refinement statistics

	*P.li.LSUI2*	*P.li.LSUI2*
	Pre-2S	Post-catalytic
*Data collection*		
Space group	*C*222_1_	*C*222_1_
Cell dimensions		
*a*, *b*, *c* (Å)	165.0, 256.9, 137.2	163.7, 255.4, 136.8
*α*, *β*, *γ* (°)	90, 90, 90	90, 90, 90
Resolution (Å)	150.00–3.70 (3.83–3.70)	150.0–3.68 (3.74–3.68)
*R*_sym_ (%)	8.6 (95.1)	14.9 (>100)
*I* / *σI*	15.1 (1.21)	6.4 (0.6)
Completeness (%)	97.1 (92.9)	99.9 (99.9)
Redundancy	3.5 (3.2)	6.8 (3.8)
CC^a^	(0.926)	(0.743)
*R*_pim_ (%)	5.3 (58.8)	8.5 (>100)
CC_1/2_	(0.751)	(0.381)
*Refinement*		
Resolution (Å)	50.08–3.70 (3.82–3.70)	81.82–3.68 (3.81–3.68)
No. reflections	31,200	31,107
*R*_work_ / *R*_free_	21.73/25.29 (32.36/33.97)	20.81/25.52 (36.50/41.78)
No. atoms	13,884	14,107
RNA	13,543	13,479
Ligand/ion	104	382
Water	236	246
*B*-factors		
RNA	207.5	197.6
Ligand/ion	187.3	198.3
Water	137.1	159.0
R.m.s. deviations		
Bond lengths (Å)	0.002	0.010
Bond angles (°)	0.515	1.165

^a^Values in parentheses are for highest-resolution shell.

**Fig. 2:**
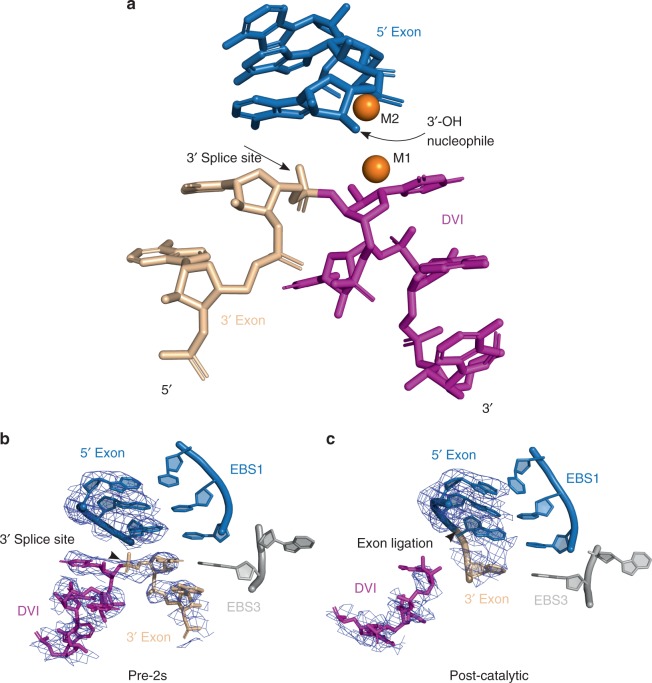
Configuration and density for the 3′ splice site in the lariat 3′-exon (pre-2s) intermediate. **a** Close-up view of the stereochemistry of the 3′ splice site and its close proximity to the catalytic metal ions M1 and M2, as well the 3′-OH nucleophile of the 5′ exon. Feature-enhanced maps (contoured at 1*σ*) were calculated for the region spanning the 3′ splice site in both the pre-2s (**b**) and post-catalytic (**c**) states. Density for the cleaved 3′ end is clearly visible in the post-catalytic state versus the intact 3′ splice site in the pre-2s structure

Examination of the intron core reveals the 3′-OH of the 5′ exon poised to engage in nucleophilic attack at the intact 3′ splice site (Fig. 2a). The scissile phosphate junction at the 3′ splice site is highly distorted and kinked as predicted by Chan et al. (2012)^11^. This distortion likely aids in presenting the correct scissile phosphate to the catalytic metal ions for cleavage in the second step in addition to applying strain on this bond to assist cleavage. In order to reduce model bias, feature-enhanced maps^12^ were calculated for nucleotides spanning the 3′ splice site for both the pre-2s and post-catalytic structures (Fig. 2b, c). This reveals density for an intact 3′ splice site in the pre-2s crystals and confirms that this structure represents the state directly preceding the second step. A comparison of both structures reveals significant differences within the catalytic domain V, the branch-site helix domain VI and the 3′ end of the intron.

γ-γ′ is not engaged in the observed pre-2s state, thus accounting for the 10 Å distance between the 3′ splice site and the catalytic metal ions M1 and M2. The γ nucleotide from J2/3 is flipped 180° away from the active site and therefore cannot pair with the 3′ terminal nucleotide (γ′) of the intron. The lack of this interaction accounts for the fact that the 3′ splice site is intact in this structure. This also suggests that γ–γ′ is a transient and dynamic contact that forms during the cleavage of the 3′ splice site (see below). Therefore, further conformational changes are required in order for this active site to engage in the chemistry required for the second step of splicing.

**Fig. 3:**
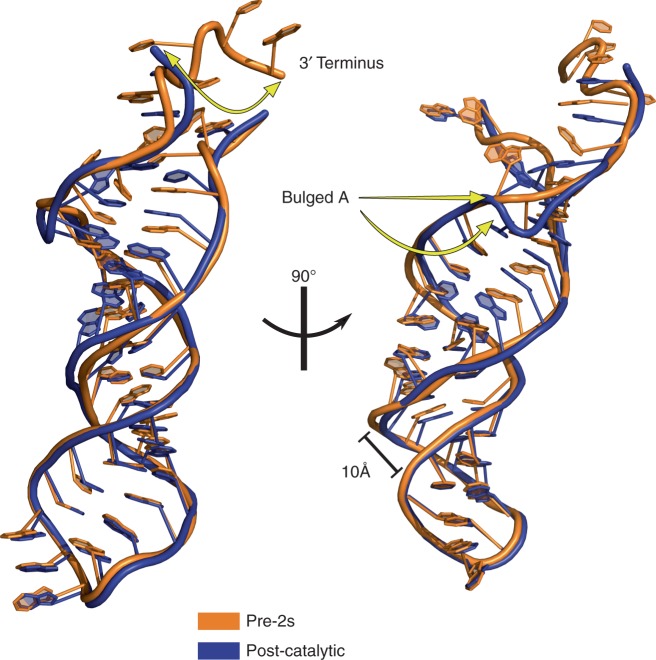
Superimposed structures of DVI in the post-catalytic and pre-2s states. This reveals conformational rearrangements at the basal region of the DVI stem that are responsible for 3′ splice site positioning. This basal region undergoes a local compression in the transition from the pre-2s state to exon ligation. During this transition, the backbone of the bulged adenosine residue is distorted in the post-catalytic state

A comparison of the pre-2s and post-catalytic structures reveals that the basal stem of domain VI, which contains the branch-point adenosine, undergoes a compression in the transition to the second step. Figure 3 shows a superposition of the domain VI region from both structures. In particular, the backbone of the bulged adenosine residue is distorted in the post-catalytic state upon the completion of exon ligation. Given that the scissile phosphate of the 3′ splice site is not in a position to be cleaved in the pre-2s structure, we hypothesize that this compression of the domain VI helix assists in positioning the 3′ splice site in close proximity to the catalytic metals ions and the 3′-OH nucleophile of the 5′ exon for ligation.

This observed compression of domain VI is likely not the result of crystal packing interactions as this region is relatively free of crystal contacts in the lattice (Supplementary Figure 3). In addition, the space group and unit cell dimensions remain essentially unchanged between the two states, which is further evidence that these conformational rearrangements are not the result of alterations in crystal packing in the presence and absence of iridium hexammine.

### Catalytic triplex undergoes conformational rearrangements

**Fig. 4:**
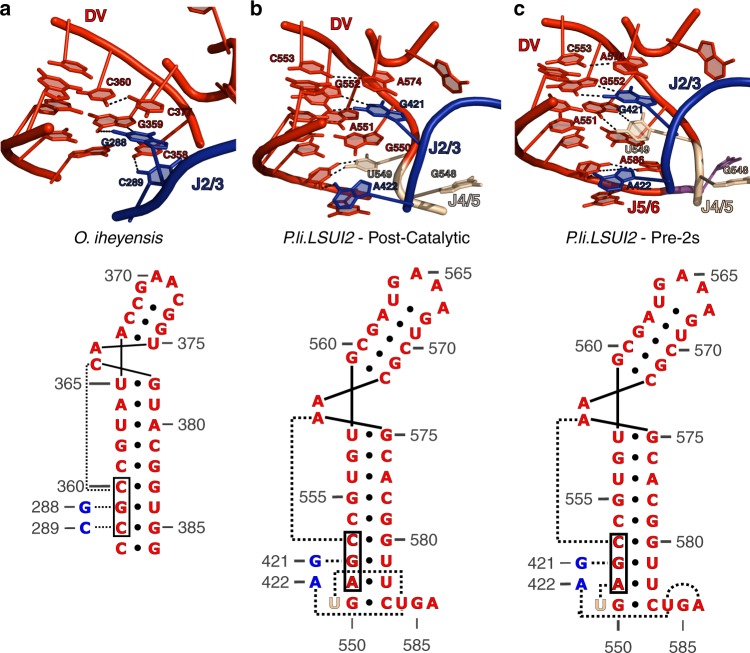
Different configurations of the catalytic triplex. This shows the three known configurations of the catalytic triplex in group II introns. The configuration in *O. iheyensis* (**a**) is thought to represent the configuration required for the first step. **b**, **c** represent the pre-2s and post-catalytic states of the *P.li.LSUI2* intron, respectively. Unlike *O. iheyensis*, J4/5 and J5/6 are involved in forming base triples in *P.li.LSUI2*. These base triples rearrange during the second step of splicing. The secondary structure representations of the base triple patterns are directly below the corresponding three-dimensional structure. The colors represent the different domains of the intron as shown in the secondary structure in Fig. 1

The catalytic triplex in group II introns consists of consecutive base triples involving nucleotides from the catalytic triad, the two-nucleotide bulge, and J2/3 (Fig. 4a). Marcia and Pyle (2012) were the first to discover the existence of conformational rearrangements in the catalytic triplex of the bacterial group IIC intron during the first step of hydrolytic splicing^13^. These rearrangements were observed through growth of the crystals in ionic conditions not supporting splicing. This work revealed that J2/3 is disengaged from the catalytic triad in the pre-catalytic state and is engaged in the active form. Therefore, this suggests that the residues involved in the catalytic triplex are dynamic during splicing.

The structure of the post-catalytic eukaryotic IIB intron revealed the first evidence for additional junction nucleotides (other than J2/3) participating in base triples within the catalytic triplex^10^. Specifically, the junctions between domains IV and V (J4/5) and domains V and VI (J5/6) are also involved in forming triples at the base of domain V (Fig. 4b). This is a different triplex configuration compared to that seen in IIC introns (Supplementary Table 1). It is likely that the IIC intron represents the basal triplex configuration required for the first step because it is missing the 3′ splice site, J5/6, and domain VI, and therefore cannot catalyze exon ligation. In comparison with the IIC intron, we observed in the post-catalytic IIB intron that A422 of J2/3 disengages with A551 of the catalytic triad to form a base triple with U549 of J4/5 and U584 of J5/6 at the base of domain V. Surprisingly, the pre-2s structure presented in the current study reveals yet another configuration of the catalytic triplex. In contrast to the post-catalytic structure, A586 of J5/6 has taken the place of U549 in the base triple below domain V and U549 is observed stacking underneath the nucleobase of the J2/3 residue G421 (Fig. 4c). We hypothesize that this catalytic triplex represents an active conformation that corresponds to the second step of splicing.

### Mutagenesis supports dynamic catalytic triplex

**Fig. 5:**
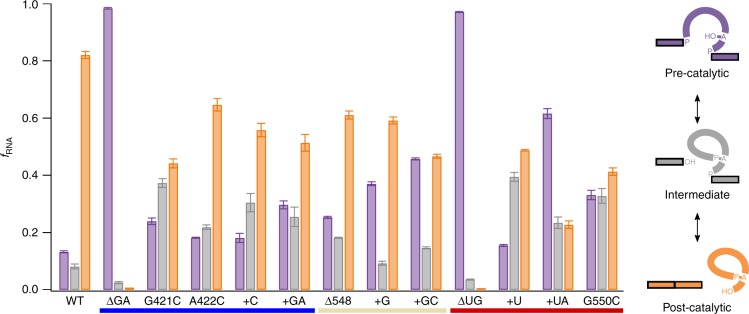
Splicing assays of junction sequence mutants. Fraction of precursor (purple), linear intron-3′ exon with lariat-3′ exon (gray), and linear intron with lariat (orange) are shown for wild type and several mutations of key residues in J2/3 (blue underline), J4/5 (wheat underline), or J5/6 (red underline). The ΔUG mutation of J5/6 was the only mutation observed to alter the preference of the first step nucleophile as this mutation results in a complete loss of branched product. All assays were done in triplicate with the error bars representing the standard deviation at each time point

To test the function of the junction sequences, we altered the sequence and length of J2/3, J4/5 and J5/6 (Fig. 5). Deletion of the conserved GA sequence within J2/3 results in a severe catalytic defect with almost no splicing, which is expected given the importance of the catalytic triplex in active site formation. However, insertion and point mutants of J2/3 affect both the first and second steps depending on the specific mutation. For example, G421C exhibits a 4.5-fold greater accumulation of lariat-3′ exon intermediate compared to WT. Similarly, J4/5 and J5/6 mutants also exhibit step-specific defects with the J5/6 + U mutant having a 4.8-fold accumulation of lariat-3′ exon intermediate (second step defect) and J5/6 + UA having a 4.6-fold increase in precursor (first step defect). This suggests that different mutations in these junctions can trap the intron in a conformation to favor one step over the other, which supports our hypothesis that the catalytic triplex must rearrange between the different steps of splicing. This is also consistent with previous mutagenesis experiments involving J2/3 and J5/6 in the group IIB *aI5*γ intron^14,15^.

### SHAPE-MaP analysis confirms conformational rearrangements

**Fig. 6:**
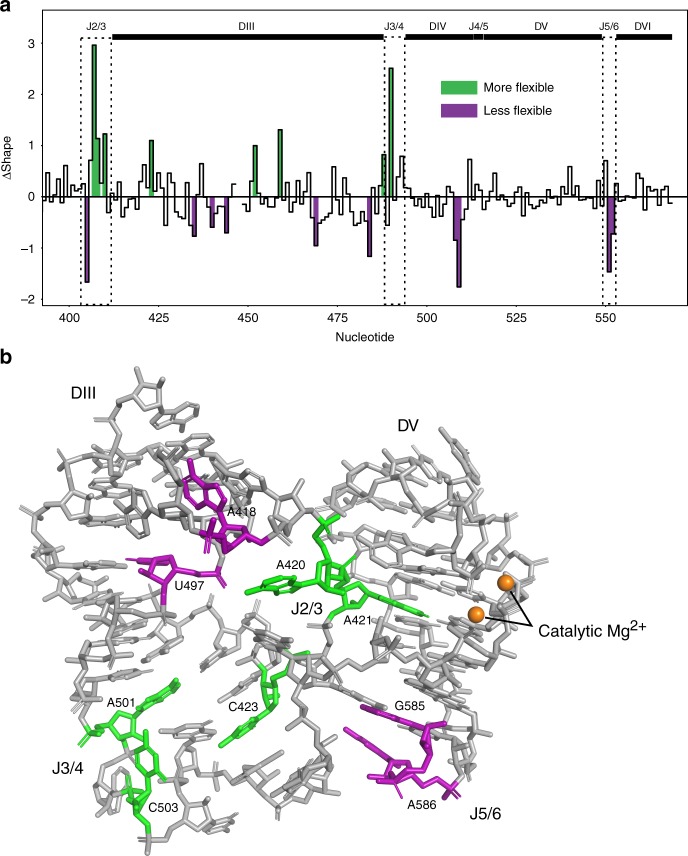
SHAPE-MaP analysis. **a** Differences in SHAPE reactivity compared between the pre-2s and post-catalytic states. Colored regions are nucleotides with significant reactivity differences (*Z*-factor > 0 and |standard score| ≥ 1 as determined by the algorithm and colored by the ΔSHAPE program). Green represents nucleotides that are more flexible in the post-catalytic form compared to the pre-2s state. Purple represents nucleotides that are less flexible in the post-catalytic compared to pre-2s. **b** Junction regions of the post-catalytic intron labeled with SHAPE difference data. Colors are the same as **a**. Only the basal stem of domain III is shown. The junction sequences are in close proximity to the catalytic Mg^2+^ ions

We performed selective 2′-hydroxyl acylation analyzed by primer extension and mutational profiling (SHAPE-MaP)^16,17^ experiments to provide additional evidence for the conformational rearrangements observed in our crystal structures. SHAPE-MaP utilizes high-throughput sequencing to allow for more sensitive detection of the reactivity of individual nucleotides to the 1M7 SHAPE reagent. This experiment directly reports on the flexibility of RNA residues in solution with single-nucleotide resolution. This analysis was done on the wild-type intron in the post-catalytic state and a mutant trapped in the pre-2s state. The SHAPE reactivity profile of the group II intron in the intermediate lariat-3′ exon conformation was subtracted from that of the post-catalytic lariat conformation to generate a ΔSHAPE profile, which reveals structural differences that occur during the second step of splicing. This data reveals that J2/3, J3/4, and J5/6 undergo dynamic rearrangements during the second step of splicing (Fig. 6a). Mapping of the SHAPE-MaP data on the structure reveals that these dynamic regions are clustered close together in three-dimensional space and are directly adjacent to the two catalytic metal ions in the active site (Fig. 6b). In contrast, J4/5 is highly modified in both the pre-2s and post-catalytic states, however there is no difference in the level of modification between these states. Nucleotides in domain III are also found to be dynamic and in close proximity to J2/3 and J3/4. This is consistent with the proposed role for domain III as an allosteric effector of catalytic activity^18^. Therefore, it is possible that domain III affects catalysis through its interactions with these junction nucleotides.

### Model for the second step of group II intron splicing

**Fig. 7:**
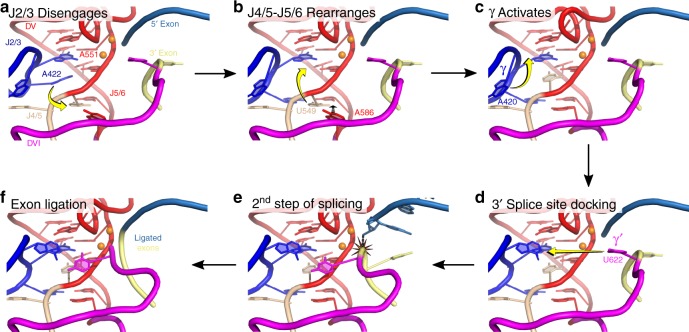
Model for the second step of group II intron splicing. This model illustrates events occurring after the first step with the exit of the 2′–5′ lariat phosphodiester bond from the active site. **a** The catalytic triplex prepares for the second step with J2/3 residue A422 disengaging from A551. **b** U549 replaces the position previously occupied by A422. **c** EBS3-IBS3 then forms to anchor the 3′ end of the intron (not shown) and causes A586 of J5/6 to displace U549 at the base of DV to activate the catalytic triplex for the second step. This creates a binding pocket for A420 (γ), which enters its active conformation by stacking under A573 and allows subsequent pairing with the 3′ splice site. **d** The formation of γ–γ′ draws the 3′ splice site near the catalytic metal ions and promotes exon ligation. **e** The 3′-OH of the 5′ exon engages in nucleophilic attack at the 3′ splice site. **f** Intron is in the post-catalytic state with ligated exons bound in the active site before subsequent release

**Fig. 8:**
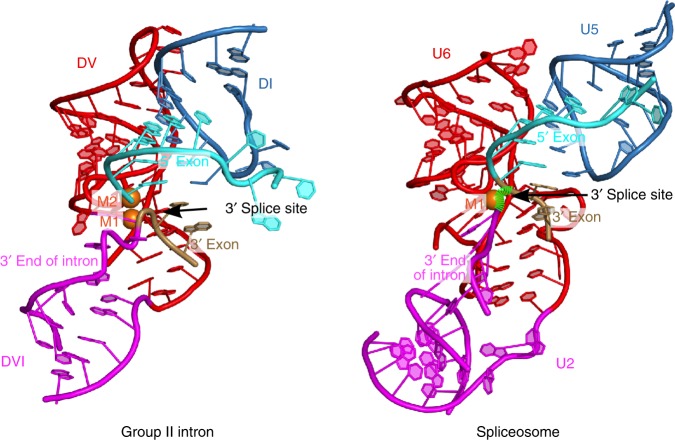
Pre-2s state vs. the spliceosomal P complex. The location of the 3′ splice site is indicated with arrows. The spliceosomal P complex (PDB accession 6EXN) contains a cleaved 3′ splice site with the green hatched lines indicating the probable backbone direction of the intact splice site given the location of the 3′ intron end and the 3′ exon. In both cases, the 3′ splice site adopts a highly kinked configuration that presents the scissile phosphate to the 5′ exon for nucleophilic attack. The overall configuration of the active sites exhibits structural homology indicating an evolutionarily conserved mechanism for the second step of RNA splicing

The cumulative data are consistent with a model in which dynamic rearrangements of the catalytic triplex are likely responsible for the positioning and removal of exon substrates from the active site in the transition between the two steps of splicing (Fig. 7). This model interpolates between the known configurations of the catalytic triplex (Fig. 4) and the location of the engaged γ–γ′ interaction. Therefore, this model is based on the crystal structures of the *P.li.LSUI2* and *O. iheyensis* introns and previous biochemical data for interactions in the active site. As a result, each panel in Fig. 8 represents a theoretical remodeling of the known *P.li.LSUI2* crystal structures that transition between the different triplex configurations and 3′ splice site placement. A central feature of this model is a series of conformational rearrangements involving the catalytic triplex that are necessary to create a binding pocket for the γ nucleotide A420. This positions A420 so that it can pair with the 3′ splice site for the second step and brings the scissile phosphate in close proximity to the catalytic metals ions for cleavage. In the first stage, J2/3 residue A422 disengages and then re-engages at a lower level within domain V. This creates an initial opening to allow formation of the γ-binding site. The binding site is completed with rearrangements in J4/5 and J5/6 creating additional base stacking interactions with the incoming γ nucleotide A420. Formation of the γ–γ′ interaction then pulls the 3′ splice site into the catalytic core for exon ligation. These conformational rearrangements can be described as a series of nucleobase flipping events within the active site to prepare for the second step of splicing.

### Comparison with chimeric *O. iheyensis* IIC intron construct

Recently, the crystal structure of a chimeric IIC intron lariat was determined^19^. This construct is a modified version of the wild-type *O. iheyensis* intron structure previously determined by Toor et al.^20^. Specifically, this construct is a chimera containing the addition of a truncated domain VI from a IIC intron found in a different bacterial species. As a result, it is lacking both a wild-type J5/6 and an intact 3′ splice site. However, this structure does show the γ–γ′ interaction in an engaged state, which is likely trapped due to the lack of an intact 3′ splice site. Furthermore, the location and conformation of the γ–γ′ interaction in this structure is consistent with our model for the second step of splicing presented above.

### Parallels with the spliceosomal P complex

A comparison of the pre-2s state with the spliceosomal P complex^7–9^ reveals homology in active site architecture and 3′ splice site substrate placement (Fig. 8). The pre-2s structure does not correspond to the spliceosomal C* complex since the 3′ splice site is not docked into the active site of the C* complex^4,21,22^. The spliceosomal P complex represents the post-catalytic state immediately after the second step of splicing. However, the 3′ end of the intron is still docked near the active site of the spliceosome and therefore provides insight into the second step. Tracing a path from the 3′ end of the intron to the beginning of the 3′ exon reveals a kinked curvature in the predicted location of the 3′ splice site. This is a similar configuration to that seen in the pre-2s state of the group II intron. Therefore, this splice site distortion is likely a conserved feature of both group II introns and the spliceosome and further extends the parallels between the two splicing systems.

Alternative catalytic triplex configurations have not yet been observed in the cryo-EM structures of the spliceosome to date. One possible explanation is that alternative triplex configurations have not been trapped into a homogeneous state with cryo-EM which typically captures and classifies heterogeneous structures on the basis of large-scale differences. As a result, multiple conformations at the individual nucleotide level may be averaged. In contrast, RNA crystallization results in a more homogeneous population that can enable these small-scale distinctions to be more easily visualized with the use of *F*_o_*-F*_c_ omit maps. A second possibility is that alternative triplexes in the spliceosome are transient during catalysis and revert to the ground state configuration. In this case, future SHAPE-MaP probing of the spliceosomal catalytic triplex may reveal these dynamics. Yet another explanation is that these triplex dynamics may have been replaced by RNA-protein interactions in the spliceosome to accomplish an analogous role. Mutagenesis of RNA-protein interfaces in the spliceosomal catalytic core may provide evidence for this alternative. Therefore, the study of group II introns can guide the design of further experiments to probe the catalytic mechanism of the spliceosome.

## Methods

### Structure determination

The pre-2s state of the *P.li.LSUI2* intron was crystallized in sitting drops by vapor diffusion at 30 °C. Equal volumes of RNA (10 mg ml^−1^) were mixed with 0.4 mM spermine, 21% 2-methyl-2,4-pentanediol (MPD), 175 mM magnesium acetate tetrahydrate, and 90 mM MES monohydrate (pH 5.6). Crystals appeared within 2 days and were gradually exchanged into 21% MPD, 100 mM magnesium acetate tetrahydrate, 50 mM MES monohydrate (pH 5.6), 3 mM iridium hexammine, 0.5 mM spermine, and 100 mM NaCl, followed by flash freezing in liquid nitrogen. Post-crystallization treatment was altered to exclude iridium hexammine. X-ray data sets were collected at NE-CAT’s 24-ID-C beamline at the Advanced Photon Source (Argonne National Laboratory, Argonne, Illinois). Data was processed using HKL-2000^23^. The post-catalytic structure of *P.li.LSUI2* (PDBID 4R0D) was used as a search model for molecular replacement using PHENIX^24^. The last 10 and 5 nucleotides of the intron and 5′ exon, respectively, were deleted to avoid model bias at the splice sites. RNA nucleotides were modeled using COOT^25,26^ and the RCrane plugin^27,28^. Structure refinement was done using Buster^29^, PHENIX, DEN^30^, and Phenix.Erasser^31^. Feature-enhanced maps^12^ were calculated using PHENIX. SBGrid compiled all the software used^32^.

### In vitro **self-splicing assays**

Constructs used for in vitro self-splicing assays were modifications of the wild-type (WT) *P.li.LSUI2* sequence with the DIV ORF removed and a 250 nt 5′ exon and 75 nt 3′ exon. This construct was cloned into pUC57 using the EcoRV cut site. DNA templates for in vitro transcription were linearized using BamHI. Radiolabeled transcripts were prepared using T7 RNA polymerase, 5 mM MgCl_2_, 40 mM Tris-HCl pH 7.5, 0.05% Triton X-100, 10 μCi [α-^31^P]UTP (3000 Ci mmol^−^^1^), 0.5 mM UTP, and 1 mM other NTPs. In vitro transcription reactions were done for 1 h at 37 °C followed by purification of the precursor RNA on a denaturing 4% (19:1) polyacrylamide, 8 M urea, 1X TBE gel. Radiolabeled precursor RNA was refolded in 40 mM Tris-HCl pH 7.5 through heating at 90 °C for 1 min followed by incubation in 40 mM Tris-HCl pH 7.5, 10 mM MgCl_2_ for 15 min. To initiate self-splicing, RNA was mixed with an equal volume of 2X splicing buffer (2 M NH_4_Cl, 40 mM Tris-HCl pH 7.5). Reactions were quenched by mixing with an equal volume of 80% formamide, 100 mM EDTA. Splicing products were resolved using denaturing 4% (19:1) polyacrylamide, 8 M urea, 1× TBE gels. All splicing assays were done in triplicate.

### Quantitation of self-splicing products

Splicing gels were exposed to storage phosphor screens and splicing products were quantitated using Quantity One 1-D Analysis Software. Unequal loading was accounted for by normalizing to an internal control RNA. Band intensity was determined by dividing the background-subtracted intensity of each band by the number of uridine residues in the RNA sequence corresponding to the band. All band intensities were then normalized to an unspliced control to give fractional values of input RNA.

### 3′ end mapping

Native and Ir(NH_3_)_6_^3+^-soaked crystals were rinsed with excess crystallization solution to remove uncrystallized RNA then dissolved in 50 mM HEPES pH 7, 100 mM NaCl, 1 mM DTT, 0.1 μM *Entamoeba histolytica* Dbr1 debranching enzyme^33^ and incubated at 37 °C for 30 min to hydrolyze the lariat bond. RNA was phenol-chloroform extracted, ethanol precipitated and resuspended in nuclease free water. One microgram debranched RNA was incubated with 1 mM ATP and 5 units of *E. coli* poly(A) polymerase from NEB at 37 °C for 30 min to add a poly(A) tail to the RNA. The RNA was phenol-chloroform extracted, ethanol precipitated and resuspended in nuclease free water. 200 ng oligo(dT)_18_ and 1 μL 10 mM dNTP mix were added to the RNA and the volume was brought to 13 μL with nuclease free water. To anneal the primer, the mixture was heated at 65 °C for 5 min and incubated on ice for 1 min. The RNA was reverse transcribed at 50 °C for 30 min using 200 units SuperScript^TM^ III RT, 1X first-strand buffer, and 5 mM DTT in a final volume of 20 μL, and the RT was inactivated by heating at 70 °C for 15 min. Two microliter of cDNA was used as a template for PCR using oligo(dT)_18_ as a reverse primer and a forward primer that anneals within the intron. PCR products containing a short poly(A) tail were gel extracted, cloned into pUC57 using the EcoRV cut site, and transformed into *E. coli* to sequence single colonies. This 3′ end mapping protocol is derived from that described by Qin et al. (2016)^34^.

### SHAPE-MaP analysis

A second-step deficient *P.li.LSUI2* group II intron was constructed by mutating G to A in EBS3 and U to A in γ′. Lariat-3′ exon and post-catalytic group II intron RNAs were prepared by the non-denaturing purification method^20,35^. Lariat-3′ exon and post-catalytic RNA was stored in 10 mM MgCl_2_ and 5 mM sodium cacodylate pH 6.5. The RNAs were chemically modified with 1-methyl-7-nitroisatoic anhydride (1M7) using the small RNA workflow of the SHAPE-MaP protocol^17^. The reverse transcription primer hybridizes to the 3′ exon (lariat-3′ exon) or the 3′ end of the intron (post-catalytic). Primers were designed to amplify about 500 nucleotides of the cDNA. Libraries were sequenced on a MiSeq with a 2 × 300 basepair paired-end configuration. Differences in SHAPE reactivities were analyzed with the ΔSHAPE script^16^. Using the ΔSHAPE program^16^, the reactivities of the pre-2s mutant were subtracted from the post-catalytic state. Significant differences at a given nucleotide position were determined by applying the following statistical criteria:

1 $$Z - {\rm{factor}}_{\it{i}} = 1 - \frac{{1.96(\sigma _{i,A} + \sigma _{i,B})}}{{\left| {\Delta {\rm{SHAPE}}_i} \right|}} > \hskip 2pt 0$$

2 $$\left| {{\rm{Standard \hskip 4pt score}}_i} \right| = \left| {\frac{{\Delta {\rm{SHAPE}}_i - \mu _{\Delta {\rm{SHAPE}}}}}{{\sigma _{\Delta {\rm{SHAPE}}}}}} \right| \ge 1$$

The Z-factor criteria establishes that the 95% confidence intervals of the SHAPE reactivity measurements do not overlap. The standard score criteria returns only nucleotides with differences greater than one standard deviation from the mean of all the differences. The ΔSHAPE program returns a SHAPE difference profile where the colored regions represent nucleotides with significant differences (using the statistical criteria above), where the green represents nucleotides that become more flexible in the post-catalytic state and purple represents nucleotides that become more structured. Therefore, the ΔSHAPE profile highlights nucleotides that experience conformational changes during the second step of splicing.

## Electronic supplementary material

Supplementary Information

## Data Availability

The coordinates and structure factor data for the pre-2s and post-catalytic structures have been deposited in the Protein Data Bank (PDB) with accession codes 6CHR and 6CIH, respectively. All other data that support the findings of this study are available from the corresponding author upon reasonable request.
