# Supplementary material for: Structural basis for the second step of group II intron splicing
Source: Nat Commun. 2018 Nov 8;9:4676. doi: 10.1038/s41467-018-06678-0 (PMC6224600; doi:10.1038/s41467-018-06678-0)
Supplement: Supplementary file 1 — Supplementary Information [file 41467_2018_6678_MOESM1_ESM.pdf]

Structural basis for the second step of group II intron splicing

Russell T. Chan, Jessica K. Peters *et al.*

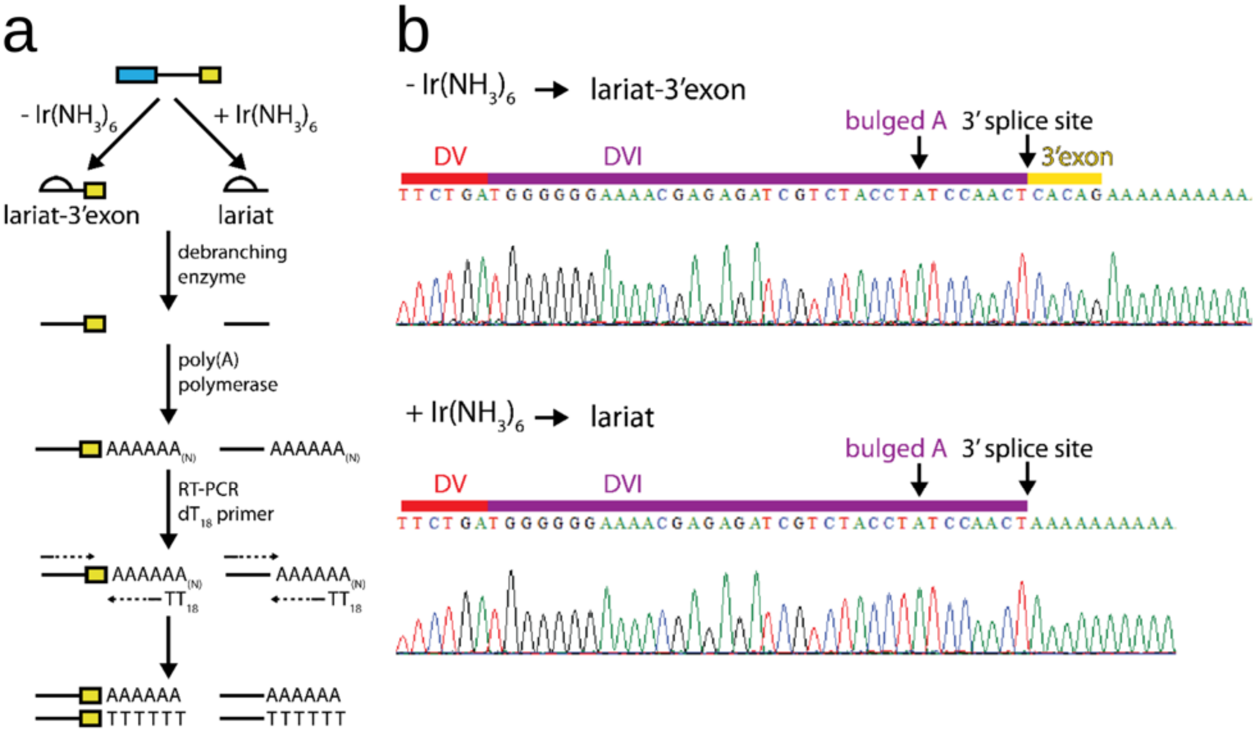

Supplementary Figure 1: Sequence analysis of the 3′ end of crystallized RNA from the pre-2s and post-catalytic states. (a) Crystallized RNA was first incubated with debranching enzyme to digest the 2′-5′ phosphodiester of the lariat. This allows 3′ end mapping without steric clashes with the reverse transcriptase and the lariat bond during primer extension. A poly(A) tail was added to the 3′ end using poly(A) polymerase. Primer extension was done using an oligo dT primer and reverse transcriptase. **(b)** Fluorescent sequencing traces show an intact 3′ splice site in the absence of iridium hexammine, whereas the iridium hexammine-containing sample does not contain 3′ exon. This is consistent with the x-ray data, which shows density for the 3′ splice site in the absence of iridium hexammine.

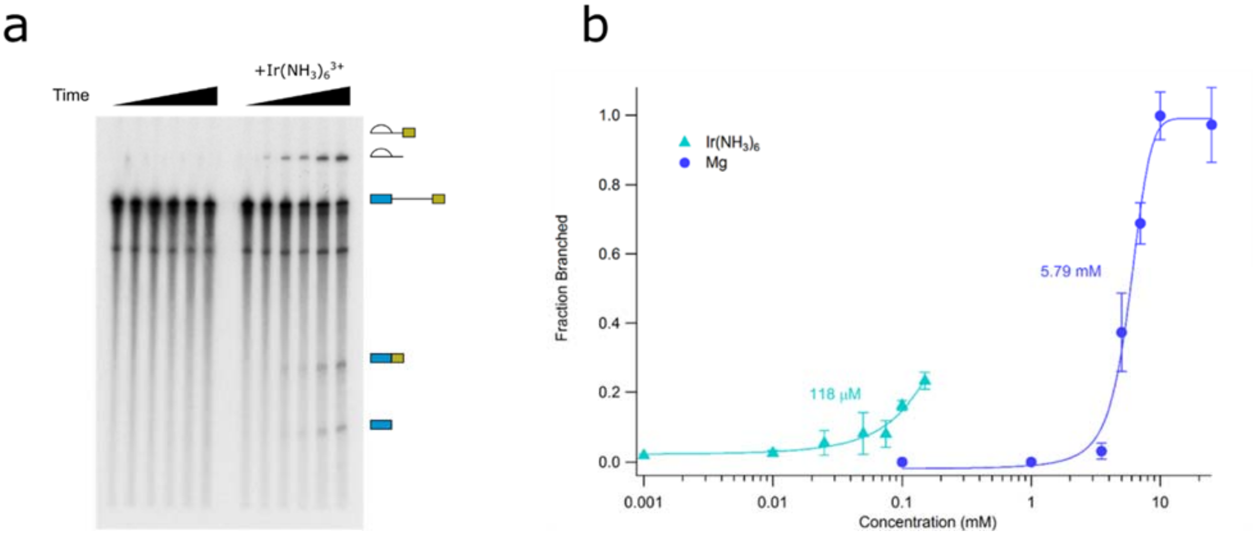

Supplementary Figure 2: Iridium hexammine stimulates the forward splicing reaction. (a) Under a limiting magnesium concentration of 3 mM, *P.li.LSUI2* exhibits no splicing activity after 2 hrs. The addition of 50 µM iridium hexammine strongly favors the post-catalytic state, with no visible pre-2s intermediate during the splicing time course. **(b)** Iridium hexammine stimulates splicing at micromolar concentrations whereas millimolar amounts of Mg^{2+} are needed to stimulate splicing. Increasing the iridium hexammine concentration to greater than 150 µM results in an inhibition of splicing. Splicing assays were done in triplicate and the error bars represent the standard deviation.

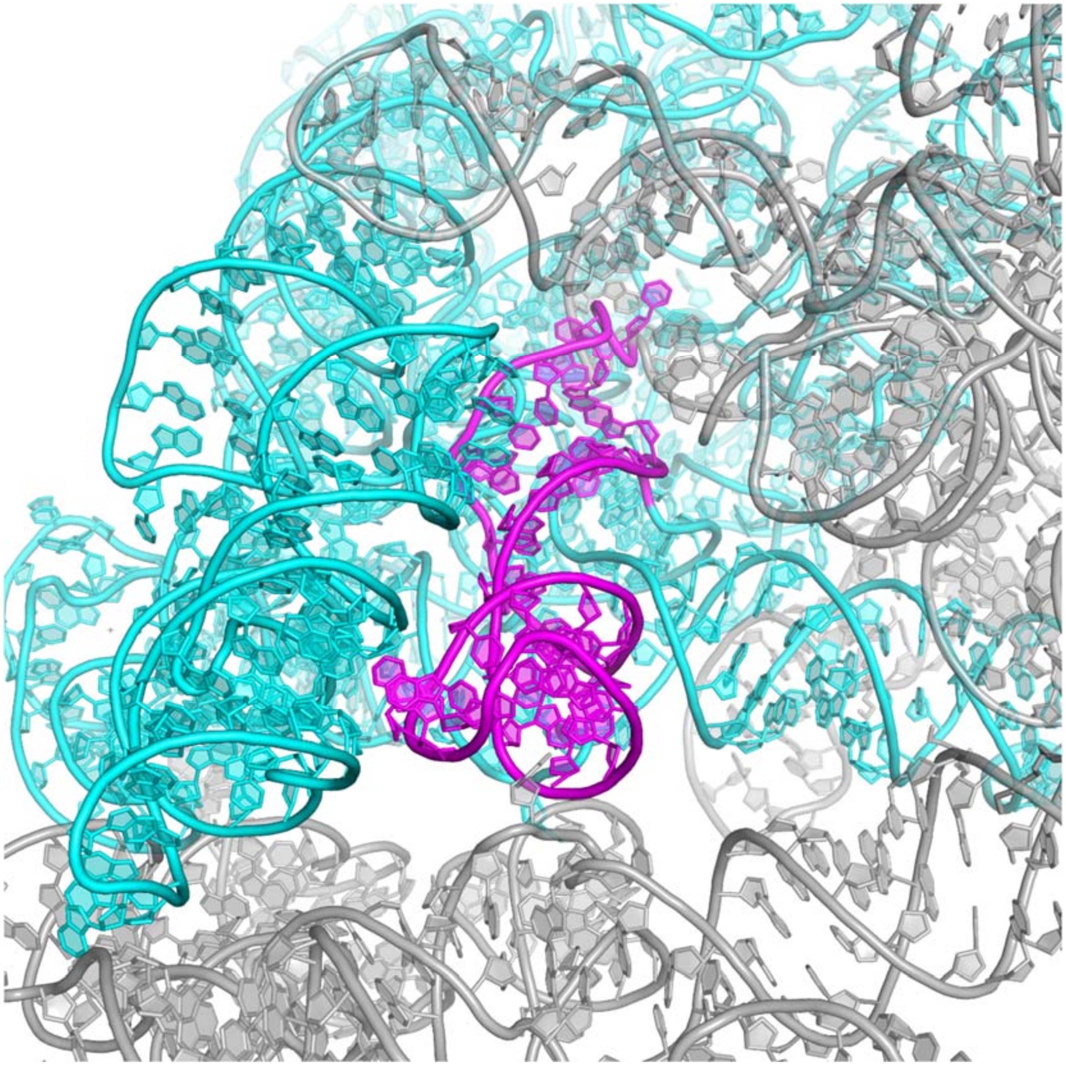

Supplementary Figure 3. Crystal packing interactions near domain VI. This shows that domain VI (purple) is relatively free of constraining crystal packing interactions from symmetry mates (gray) in the lattice.

	PDB ID	Position 1	Position 2	Position 3
O. iheyensis (IIC)	3IGI	C377	C360	G383
		G288	G359	U384
		C289	C358	G385
Spliceosomal P Complex	6EXN	(U6)U80	C61	(U2)G21
		(U6)G52	G60	(U2)C22
		(U6)A53	A59	(U2)U23
P.li.LSUI2 (IIB) pre-2s	6CHR	A574	C553	G580
		G421	G552	U581
		U549	A551	U582
		A422	A586	U584
P.li.LSUI2 (IIB) post-catalytic	6CIH	A574	C553	G580
		G421	G552	U581
		---	A551	U582
		A422	U549	U584

Supplementary Table 1: Summary of base triple configurations in group II intron structures. Boxed base triples form equivalent interactions with the catalytic triad. Other base triples are found outside of the triad in directly adjacent nucleotides in domain V.

Supplementary Note 1:

Iridium hexammine favors the post-catalytic state

Previously, we solved the structure of the *P.li.LSUI2* intron in the post-catalytic state using crystals which were soaked in iridium hexammine prior to data collection[1]. Inspection of the major groove of domain VI revealed two iridium hexammine molecules and a magnesium hexahydrate near the proposed conformational switch known as the π-π′ interaction. This interaction engages after the first step of splicing to remove the newly formed lariat out of the active site to allow entry of the 3′ splice site, which is crucial for the transition to the second step of splicing. We had postulated that the region of DVI surrounding the π-π′ interaction is flexible and dynamic to allow the observed 20 Å displacement of the lariat bond out of the active site after the first step. Data was also collected on the native dataset in the absence of iridium hexammine. Rigid body refinement of the iridium hexammine derived structure into the native dataset revealed the presence of multiple positive and negative F_o-F_c peaks within the catalytic triplex of DV and at the 3′ end of the intron (not including the absence of iridium hexammine and minor metal ions). Further analysis showed strong density corresponding to an intact 3′ splice site. In contrast, the iridium hexammine derivative had density for ligated exons in the active site. Therefore, we hypothesized that the native dataset represents the lariat-3′ exon form of the intron immediately preceding the second step of splicing. Further evidence for this hypothesis was provided by an analysis of the 3′ end of the RNA present in both the native crystals and the iridium hexammine derivative. End mapping on this crystallized RNA was done to determine the cleavage pattern at the 3′ splice site (**Supplementary Figure 1**). The RNA

from the crystals was first treated with debranching enzyme to digest the 2′-5′ phosphodiester bond of the lariat. This allows primer extension at the 3′ end without interference from this branched linkage. A poly(A) tail was then added using poly(A) polymerase followed by RT-PCR and cloning. This confirmed the presence of an intact 3′ splice site in the native crystals and spliced, post-catalytic intron in the iridium hexammine derivative.

Given that the self-splicing of group II introns is reversible, we hypothesized that iridium hexammine inhibits the backward reaction and favors the equilibrium towards the post-catalytic-state in the crystals. This is supported by splicing reactions of *P.li.LSUI2* in the presence or absence of iridium hexammine in low salt (**Supplementary Figure 2**). Splicing is not supported under low magnesium and monovalent ion concentration. However, splicing can be promoted through an increase in magnesium or an addition of iridium hexammine. Splicing proceeds upon addition of as little as 50 μM iridium hexammine. In contrast, the addition of ~80-fold higher [Mg^{2+}] is needed to promote splicing in the absence of iridium hexammine. The low concentration of iridium hexammine required to initiate splicing suggests that it may be playing a more complex role beyond simple charge neutralization of the phosphate backbone. Additionally, analysis of the splicing products reveals that there is no visible lariat-3′ exon intermediate (representing the pre-second step state) for reactions containing iridium hexammine. This is unusual because under standard splicing conditions (absence of iridium hexammine) *P.li.LSUI2* accumulates a significant amount of lariat-3′ exon. The fact that this intermediate is not visible in the presence of iridium hexammine suggests that this metal complex has a stimulatory effect on the second step to favor the post-catalytic state.

It is possible that the iridium hexammine is compensating for a natural ligand such as a positively charged amino acid residue from the cognate maturase protein. The *P.li.LSUI2* intron

does encode an open reading frame for a full-length maturase protein in domain IV, which has been removed in the crystallization construct. Therefore, it is possible that the pre-2s structure could represent an off-pathway structure in which the γ-γ' interaction is disrupted. Future structures of the group IIB intron complexed with the maturase may reveal that the protein may modulate splicing through interactions with domain VI and other regions of the catalytic core.

Supplementary References:

1. Robart, A.R., Chan, R.T., Peters, J.K., Rajashankar, K.R. & Toor, N. Crystal structure of a eukaryotic group II intron lariat. *Nature* **514**, 193-7 (2014).
